# Measurement of mid-arm muscle circumference and prognosis in stage IV non-small cell lung cancer patients

**DOI:** 10.3892/ol.2013.1128

**Published:** 2013-01-11

**Authors:** RAFAELA FESTUGATTO TARTARI, JANE MARIA ULBRICH-KULCZYNSKI, ANTÔNIO FABIANO FERREIRA FILHO

**Affiliations:** 1 Department of Nutrition, Santa Casa de Misericórdia Hospital;; 2 Department of Pathology, Hospital de Clinicas de Porto Alegre;; 3 Department of Clinical Oncology, Oncosinos, Porto Alegre, Brazil

**Keywords:** nutritional assessment, advanced lung cancer, prognostic

## Abstract

Overall survival (OS) varies widely in patients with stage IV non-small cell lung cancer (NSCLC). Strong prognostic factors are still needed to improve decision-making regarding standard treatment options, to stratify patients for inclusion in innovative therapeutic trials and to identify patients who would be best treated with palliative care rather than with systemic chemotherapy. Mid-arm muscle circumference (MAMC) is a bedside anthropometric measurement that estimates somatic protein reserve, an early indicator of nutritional depletion. This measurement is simple, non-invasive, objective and inexpensive to perform. We evaluated MAMC as a potential prognostic factor in patients with stage IV NSCLC. A total of 56 non-selected consecutive patients with stage IV NSCLC were evaluated. The MAMC measurement results for these patients were expressed as a percentage of the expected reference values, adjusted for gender and age. Patients were categorized as normal (MAMC ≥90%) or depleted (MAMC <90%). The mean age of patients was 63 years (range 47–80), and the mean MAMC was 89 (range 66–122), with 55% of patients classified as depleted. The median OS was 6.2 months (95% CI, 5.1–7.3). In the subgroup with normal MAMC, the median OS was 10.2 months (95% CI, 9.2–11.1). In patients classified as depleted, the median OS was 5.0 months (95% CI, 4.2–5.8). The difference in OS between these two subgroups was highly significant (p<0.001 by the log-rank test; HR=0.21; 95% CI, 0.09–0.5 for patients with normal MAMC). In a multivariate analysis with Karnofsky status, age and gender as covariates, the difference in OS between the MAMC groups remained statistically significant (p<0.001, according to the Cox proportional hazards model). MAMC is a strong independent prognostic factor in stage IV NSCLC patients. Patients with MAMC <90% of the expected value had poor OS.

## Introduction

Lung cancer represents 28% of all cancer mortalities, second only to breast cancer. Each year, ∼180,000 new cases of lung cancer are diagnosed in the United States (1,2).

Patients with stage IV non-small cell lung cancer (NSCLC) have among the lowest survival rates of patients with any type of cancer, but the overall survival time in this group is very heterogeneous. The median overall survival time ranges from eight to ten months, but 25–30% of patients succumb within six months, and ∼20% survive more than 18 months after the spread of the neoplasm (3). Therefore, accurate prognostic factors are required to improve decision-making regarding standard treatment options, to stratify patients for inclusion in innovative therapeutic trials and to identify patients that would be better served by palliative care than by systemic chemotherapy. Several prognostic factors appear to influence survival time, some more concretely than others (3,4).

Malnutrition is a common co-morbidity in patients with advanced NSCLC and is a major contributor to morbidity and mortality. Several studies have reported a decrease in overall survival, of from 30–50%, in malnourished patients with advanced lung cancer (4). A less common tool to assess nutritional status is mid-arm muscle circumference (MAMC), which provides an estimate of somatic protein reserve. This measurement is an early indicator of nutritional depletion, and it is simple, non-invasive, objective and inexpensive (5).

Decisions regarding choice of treatment modality and intensity are made more complex due to the heterogeneous survival in this population. Prognostic assessment may allow a better individualization of treatments for each patient. Thus, we investigated whether MAMC may be a useful prognostic factor in patients with stage IV NSCLC.

## Patients and methods

### Study design

Fifty-nine consecutive patients were identified as potential participants in this prospective study. Of these patients, only three declined to participate. The study was approved by the local ethics committee of Santa Casa de Misericórdia Hospital of Porto Alegre, Brazil. The inclusion criteria were: a) metastatic NSCLC confirmed by imaging, with more than one lesion identified; b) a pathological diagnosis of NSCLC made no more than one month before the signing of the informed consent form, and no prior specific anti-cancer treatment; c) patients must have signed the informed consent form. We performed nutritional and Karnofsky performance status (KPS) analysis immediately after the patients provided informed consent. The variables analyzed were MAMC, KPS, gender and age. Survival status was determined by telephone call. Data regarding chemotherapy treatment was retrieved by subsequent analysis of patient records.

### Nutritional and performance assessment

To evaluate MAMC, the mid-arm circumference (MAC) of the right arm was measured to the nearest centimeter with a measuring tape. Then, triceps skinfold thickness (TST), an established measure of fat stores, was measured to the nearest millimeter in the right arm using a skinfold caliper (Cescorf, Porto Alegre, Brazil) in a standard manner. Three measurements were taken for both TST and MAC, and the average values were calculated and recorded. MAMC, an established measure of muscle protein mass, was calculated from MAC and TST using a standard formula: MAMC = MAC - (3.1415 × TSF). The MAMC results were expressed as a percentage of the expected reference values, adjusted for gender and age. Patients were categorized as normal (MAMC ≥90%) or depleted (MAMC <90%) (6). KPS was used to classify the patients’ functional impairment.

### Statistical analysis

The statistical analysis was performed using SPSS software (version 17.0). Continuous variables were expressed as the means and standard deviation. Survival analyses were performed by Kaplan-Meier and log-rank tests. For inferential purposes, the MAMC was dichotomized using a threshold of 90%. Multifactorial analysis was performed using the Cox proportional hazards estimation.

## Results

A slight majority of the 56 NSCLC patients included in this study were female (52%), and the mean age was 63 years (range, 47–80).

Most patients had adenocarcinoma (76%), and the remaining patients had squamous cell carcinoma. Further descriptive characteristics of this sample are presented in Table I.

**Figure 1:**
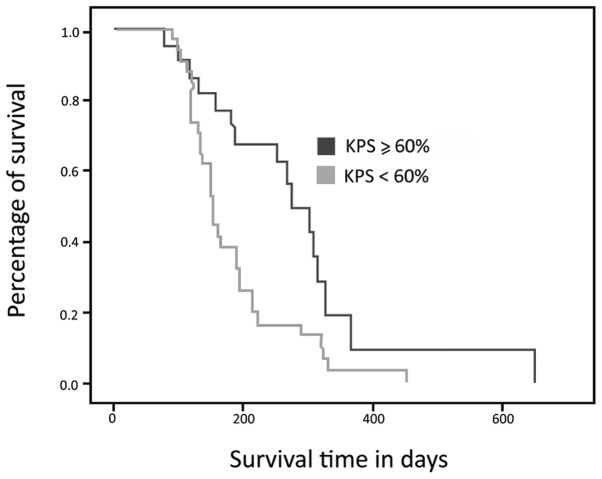
Kaplan-Meier survival curve based on Karnofsky performance status (KPS). Log-rank test, p=0.006.

Survival curve analysis showed that the survival time of the group of patients with a KPS <60% was lower than that of the group with a KPS ≥60% (Fig. 1). The patients with a KPS <60% had a median survival of 187 days (95% CI, 153–221), while the other group had a median survival of 273 days (95% CI, 215–331); this difference was statistically significant (p= 0.006). The mean KPS in this sample was 52.5%. No difference in KPS between genders was observed (p= 0.36).

**Figure 2:**
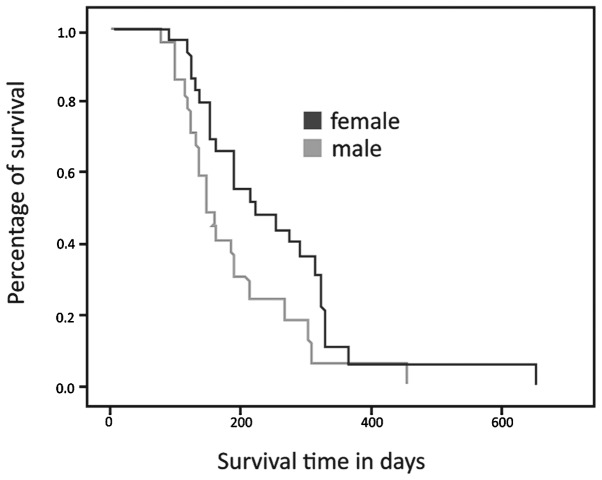
Kaplan-Meier survival curve based on gender. Log-rank test, p= 0.029.

Fig. 2 shows the survival curves according to gender. Female patients had a median survival of 221 days (95% CI, 139–302), while the males had a median survival of 149 days (95% CI, 153–220). This difference was statistically significant (p= 0.029).

The majority (55%) of patients had subnormal MAMC measurements. Malnourishment was more common in male patients (74%) than in female (p=0.014).

**Figure 3:**
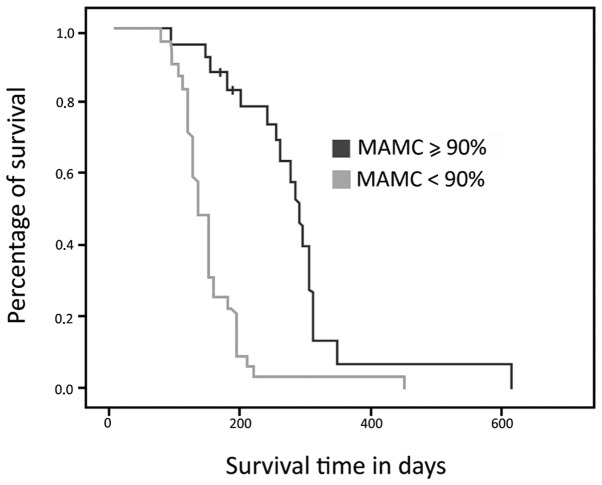
Kaplan-Meier survival curve based on MAMC. Log-rank test, p= 0.001.

Fig. 3 demonstrates that the patients identified as malnourished based on MAMC had shorter overall survival times compared to the non-malnourished group. The median survival time was 137 days in the malnourished group, (95% CI, 119–155) and 306 days in the non-malnourished group (95% CI, 278–333; p=0.001).

Table II shows the results of multivariate Cox regression analysis. Multivariate Cox modeling, after adjusting for gender, use of chemotherapy, KPS and MAMC showed that only MAMC and the use of chemotherapy were statistically significant (p<0.001; HR=0.2; 95% CI, 0.082–0.48).

## Discussion

The incidence of lung cancer has increased since the early twentieth century, when the disease was uncommon. The onset of lung cancer typically occurs in patients aged 50–70, but due to cigarette smoking, lung cancer may also be diagnosed in younger people (7). The patients in our study had an average age of 63, and the youngest patient was 47 years old. Several studies have shown an increase in the incidence of younger patients with lung cancer (3,7).

We observed a greater prevalence of lung cancer among females. The literature has shown a gradual increase in the incidence of this disease in women; however, a greater proportion of the patients with this malignancy are male. This increased incidence in women may be attributed to smoking, which is becoming increasingly common among women. Tobacco consumption was not evaluated in this study. However, the increased incidence among females is likely reflected by the predominance of adenocarcinoma (76%), the histological type more prevalent in females (8–10).

The treatment of neoplastic disease requires professional knowledge of several variables that may be involved in the evolutionary history of tumors. Prediction of survival is, perhaps, the most important piece of information to offer patients after diagnosis. However, prognostic analysis in cancer is also considered essential for defining protocols and treatment strategies that are appropriate for the clinical condition of the individual patient and for monitoring and evaluating the efficacy of the treatment (11).

KPS has always been among the main determinants of survival in patients with advanced lung cancer (7,11–15). In the present study, KPS was shown to be a prognostic factor only in univariate analysis, and it did not act as an independent determinant of overall survival in our multivariate analyses. This may be explained by the sample size, which did not allow the effect of this variable to reach statistical significance.

The data with respect to differential survival in men and women are controversial. Several papers have demonstrated better overall survival in women, while others show no difference (16–18). Certain authors consider female gender to be a positive factor for survival due to the presence of steroid receptors in patients with lung cancer (18).

Weight loss is common in cancer patients and is often a symptom already present at diagnosis. A high prevalence of weight loss is found in individuals with lung and gastrointestinal tumors. Several studies have shown that weight loss is an independent predictor of survival in patients with cancer, and it has been associated with poorer physical function, increased psychological distress and reduced quality of life (19–21).

Moreover, patients with advanced lung cancer are distinct in that a marked loss of lean body mass does not typically occur in patients with weight loss due to other causes. A significant loss of lean body mass is one diagnostic factor for anorexia cachexia syndrome (ACS), which occurs in ∼50–70% of patients in this population (22).

High rates of total organic protein synthesis and turnover and metabolic abnormalities in muscle protein catabolism are commonly observed in patients with advanced cancer (23). One of the manifestations of these phenomena is the atrophy of skeletal muscle and visceral organs and hypoalbuminemia (24). Decreased physical activity in cachectic patients contributes to the suppression of protein synthesis, further favoring muscle catabolism (25).

Studies have shown that protein depletion predisposes patients to inadequate wound repair, increases their susceptibility to infections and leads to weakness and reduced functional capacity. Biochemically, a loss of body protein is associated with increased serum levels of proteolysis-inducing factor (PIF), which is able to induce degradation and inhibit protein synthesis in skeletal muscle.

PIF is present in the urine of cachectic cancer patients with marked weight loss but not in patients with little weight loss. PIF is not observed in malnourished patients without tumors, in whom the mechanisms that protect muscle tissue from catabolism are still functional (23,26). Furthermore, the function of the respiratory muscles may also be specifically affected in lung cancer (as well as in other lung diseases) due to the deficit in muscle mass, worsening the quality of life for patients (26).

In the present study, we observed that the majority (55%) of patients had abnormally low MAMC measurements. By contrast, another study that evaluated the MAMC in patients with advanced lung cancer demonstrated a lower prevalence of malnutrition (∼20%). It is important to note that this study was conducted in the Canary Islands, and each population has a specific classification for MAMC (27).

A higher rate of protein malnutrition was observed in males, whereas >60% of female patients were well-nourished. This phenomenon has also been demonstrated in other studies, but there is no clear explanation in the literature (10,28,29). A hypothesis given in one of these studies is that females are generally heavier for their height than males and therefore take longer to reach the levels of malnutrition and depletion of lean body mass (30). However, weight loss was not assessed in our study.

We also observed a between-gender difference in the MAMC, but not in KPS. This suggests that the longer survival in women may be explained by the MAMC and not by KPS, as women had a smaller muscle deficit.

The studies in the literature that objectively evaluate nutritional status as a prognostic factor used measures of nutritional status other than MAMC (21,22,27–29). However, one of these studies evaluated the association of overall survival with nutritional status as assessed by the TST and MAC (31). In this particular study, the author noted that patients with lung cancer who succumbed within six months of diagnosis had lower nutritional parameter values compared to those who survived for more than six months after diagnosis. However, the univariate analysis in that study showed an association between nutritional status and poor prognosis, whereas multivariate analysis did not confirm the prognostic utility of these parameters. Furthermore, the authors did not stratify the group by disease stage.

In the current study, there was a difference of almost six months in the survival time between the eutrophic and depleted population. MAMC may thus be considered a strong prognostic factor. Moreover, MAMC measurement is non-invasive, simple, painless, inexpensive and requires little to no preparation or patient discomfort. In addition, MAMC remained a statistically significant prognostic factor when analyzing overall survival in a multivariate model, even after adjusting for gender, use of chemotherapy, KPS and MAMC.

In conclusion, when evaluating various clinical characteristics of patients with advanced NSCLC, we observed that MAMC may be a valuable auxiliary tool for prognosis as an indicator of proteolysis and cachexia.

Due to the heterogeneous survival of patients with advanced NSCLC, methods to predict survival are particularly important to allow proper selection of treatment. No diagnostic or therapeutic method for predicting outcomes in this population is yet considered the gold standard, but may be related to multiple patient characteristics such as age, clinical performance and gender, among others.

The importance of prognosis extends beyond answering the question of survival time. Accurate prognosis is likely to provide better matching of anticancer therapies, generating improved quality of life and even promoting the more appropriate selection of patients for research in developing new treatments. Further development of this framework should provide more relevant and appropriate clinical management. The identification of the patient profile is undoubtedly the optimum strategy with which to address the challenge of the individualization of treatment.

## Figures and Tables

**Table I:** Baseline characteristics.

Variables	Mean ± standard deviation
Age (years)	63±8
MAMC (mm)	22.3±2.8
MAMC adequacy (%)	89±13
KPS (%)	56±14
Overall survival (days)	187±17
Overall survival (months)	6.23± 0.56

MAMC, mid-arm muscle circumference; KPS, Karnofsky performance status.

**Table II:** Multivariate Cox proportional hazard model.

	P-value	HR	95% CI
KPS	0.800	1.102	0.519–2.341
MAMC adequacy	<0.001	0.200	0.082–0.487
Gender	0.803	1.090	0.553–2.148
Use of chemotherapy	<0.001	16.309	5.19–51.23

MAMC, mid-arm muscle circumference; KPS, Karnofsky performance status.
